# Phage Therapy as a Promising New Treatment for Lung Infection Caused by Carbapenem-Resistant *Acinetobacter baumannii* in Mice

**DOI:** 10.3389/fmicb.2017.02659

**Published:** 2018-01-09

**Authors:** Yunfen Hua, Tingting Luo, Yiqi Yang, Dong Dong, Rui Wang, Yanjun Wang, Mengsha Xu, Xiaokui Guo, Fupin Hu, Ping He

**Affiliations:** ^1^College of Pharmaceutical Sciences, Zhejiang University of Technology, Hangzhou, China; ^2^Department of Medical Microbiology and Immunology, School of Medicine, Shanghai Jiao Tong University, Shanghai, China; ^3^Institute of Antibiotics, Huashan Hospital, Shanghai Medical College, Fudan University, Shanghai, China

**Keywords:** carbapenem-resistant *Acinetobacter baumannii* (CRAB), bacteriophage, phagotherapy, lung infection, mouse model

## Abstract

Carbapenem-resistant *Acinetobacter baumannii* (CRAB) which is noted as a major pathogen associated with healthcare-associated infections has steadily developed beyond antibiotic control. Lytic bacteriophages with the characteristics of infecting and lysing specific bacteria have been used as a potential alternative to traditional antibiotics to solve multidrug-resistant bacterial infections. Here, we isolated *A. baumannii*-specific lytic phages and evaluated their potential therapeutic effect against lung infection caused by CRAB clinical strains. The combined lysis spectrum of four lytic phages’ ranges was 87.5% (42 of 48) against CRAB clinical isolates. Genome sequence and analysis indicated that phage SH-Ab15519 is a novel phage which does not contain the virulence or antibiotic resistance genes. *In vivo* study indicated that phage SH-Ab15519 administered intranasally can effectively rescue mice from lethal *A. baumannii* lung infection without deleterious side effects. Our work explores the potential use of phages as an alternative therapeutic agent against the lung infection caused by CRAB strains.

## Introduction

In the last 10 years, *Acinetobacter baumannii* has emerged as one of the major pathogens implicated in outbreaks of hospital-acquired infections, especially in patients from intensive care units (ICUs; Maragakis and Perl, 2008; Eveillard et al., 2010; Buser et al., 2017). Historically, carbapenems are the most potent and reliable β-lactam antibiotics for the treatment of serious infections caused by *A. baumannii* (Garnacho-Montero and Amaya-Villar, 2010). However, the prevalence of carbapenem-resistant *A. baumannii* (CRAB) is seriously compromising the use of carbapenems in the control of such infections (Queenan and Bush, 2007). CRAB is usually resistant to almost all available antimicrobials. Although colistin is effective against most CRAB, it is a last-resort treatment due to high toxicity. Consequently, treatment options for these infections are limited and considerable mortality is associated with CRAB (about 50%; An et al., 2017). According to CHINET surveillance from China, the average resistance rate of *A. baumannii* to carbapenems is more than 70% (Hu et al., 2016). This shows the urgent medical need to promote alternatives to fight against CRAB infections.

Bacteriophages are viruses which can specifically attack and kill their host bacteria. Phage has been applied to treat bacterial infections with therapeutic purpose by Félix d’Herelle as early as 1919 (Dublanchet and Fruciano, 2008). However, phage therapy was largely abandoned in the mid-20th century following the discovery of antibiotics (Cooper et al., 2016). Recently, due to the prevalence of antibiotics-resistant bacterial infections, phage therapy has experienced a renaissance (Oliveira et al., 2015). As an alternative to traditional antibiotic therapy, phage therapy has many advantages, such as host specificity minimal effects on the local microbiota, self-replication in host bacteria, and no critical side effects (Sarhan and Azzazy, 2015).

It is valuable to study the novel therapy strategy for CRAB pneumonia. Animal models are very useful for evaluating the efficacy of new antimicrobial agents. The cyclophosphamide-induced neutropenic mouse model of *A. baumannii* pneumonia develops a reproducible acute course of pneumonia in mice, and is commonly used to assess bacterial virulence and drug efficacy (Eveillard et al., 2010; Manepalli et al., 2013; Jacobs et al., 2014; Jeon et al., 2016). This model mimics the clinical situation in which the immunocompromised patients are at risk of developing *A. baumannii* infections. Thus, we chose the neutropenic mouse model to evaluate the phage therapy efficacy in treating *A. baumannii* infection.

In this study, we used the newly isolated lytic phage, SH-Ab15519, to treat the lethal CRAB lung infection in mice. Our results showed that phage therapy can significantly improve the mice survival rate and demonstrated that bacteriophage could be a promising candidate for controlling CRAB infections.

## Materials and Methods

### Bacterial Strains, Antimicrobial Susceptibility Testing, and Clonal Relationships

Forty-eight non-duplicate CRAB isolates were isolated between January and April in 2015 from Huashan Hospital (Fudan University, Shanghai, China), a 1300-bed tertiary-care hospital. Strain identification was performed using Vitek 2 compact system (Biomerieux, France). Among these strains, the *A. baumannii* strain 15519 was isolated from the sputum sample of an 84-year-old male patient who died from a severe pneumonia, and strain 15519 was resistant to most of the listed antibiotics in **Table 1** except colistin and tigecycline. Antimicrobial susceptibility testing was performed using the broth microdilution method recommended by the Clinical and Laboratory Standards Institute (CLSI, 2017). Breakpoint MICs of tigecycline were determined following the guidelines of the US Food and Drug Administration (with MICs ≤2 μg/ml denoting susceptibility and ≥8 μg/ml denoting resistance). Clonal relationships were analyzed by multilocus sequence typing (MLST; Adams-Haduch et al., 2011).

**Table 1:** The susceptibility of 48 *A. baumannii* to antimicrobial agents.

Antimicrobial agents	MIC range	MIC_50_	MIC_90_	Resistance (%)	Susceptible (%)
Cefoperazone–sulbactam	16–256	64	128	85.4	4.2
Ampicillin–sulbactam	16–128	64	128	93.8	0
Piperacillin–tazobactam	128 to >256	>256	>256	100	0
Ceftazidime	32–64	64	64	100	0
Cefepime	32–64	64	64	100	0
Imipenem	1–32	32	32	95.8	2.1
Meropenem	2–32	32	32	95.8	2.1
Amikacin	8 to >128	>128	>128	83.3	16.7
Gentamicin	2–64	64	64	95.8	4.2
Ciprofloxacin	16	16	16	100	0
Levofloxacin	4–32	8	32	91.7	0
Trimethoprim–sulfamethoxazole	1–16	16	16	97.9	2.1
Colistin	1–32	1	2	4.2	93.8
Tigecycline	0.25–2	0.5	1	0	100


### Isolation of Bacteriophages and Determination of Host Range

According to the enrichment technique recommended by Cui et al. (2017), we used 48 strains of *A. baumannii* clinical isolates from Huashan Hospital as host strains of phages. Lytic phages were isolated from the sewage of the Ruijin Hospital affiliated to Shanghai Jiaotong University. Briefly, 15 ml filtered sewage, 8 ml 3× Luria-Bertani broth, and 100 μl mid-log phase bacteria were mixed and kept at 37°C under shaking condition overnight. The supernatants including the phages with corresponding bacteria were incubated and purified by successive single plaque-isolation using double-layer agar method. Finally, four lytic phages were obtained and designated as SH-Ab 15519, SH-Ab 15497, SH-Ab 15708, and SH-Ab 15599. As for bacteriophage amplification, every phage with its host by certain proportion was grown in LB soft agar overlays (0.75% agar). Then the lysate was collected in SM buffer, enriched at 4°C overnight using polyethylene glycol (PEG) 8000, further purified with CsCl density gradient ultracentrifugation and stored at 4°C (Cui et al., 2017; Kwiatek et al., 2017).

The morphology of the phage was examined by a Hitachi transmission electron microscope H-9500 (Japan) according to the method described by Mendes et al. (2014). Phage titers were assessed using double-layer agar method and represented by plaque-forming unit (PFU; Merabishvili et al., 2009). The lytic spectrums of each phage against *A. baumannii* were tested by spot assay (Sasikala and Srinivasan, 2016). For combination studies, a phage cocktail containing the same concentration (10^8^ PFU/ml) of phages SH-Ab 15519, SH-Ab 15497, SH-Ab 15708, and SH-Ab 15599 was prepared and its lytic spectrum against CRAB strains was tested.

### Biological Characterization and Lytic Effect of Phage SH-Ab15519

The stability of phage SH-Ab 15519 at different pH and temperature values was checked by double-layer agar method. For thermal stability tests, phage SH-Ab 15519 suspensions (10^10^ PFU/ml) were incubated at 4, 37, 40, 50, 60, and 70°C (pH = 7.45) in SM buffer for 60 min, and then the phage titers were assessed. For pH stability tests, phage suspensions were incubated at 37°C (pH = 1, 2, 3, 4, 5, 6, 7, 7.45, 8, 9, 10, 11, or 12) in SM buffer for 60 min, respectively, and the phage titers were assessed. Phage absorption experiments were performed according to the method described by Wang J.B. et al. (2016). Phage SH-Ab 15519 and *A. baumannii* 15519 were incubated at an MOI of 0.01 for 1, 2, 3, 4, 5, 10, 15, 20, and 25 min. In every interval, the titer of unabsorbed phages in the supernatant was assayed using double-layer agar method. The free phage proportion was the amount of non-adsorbed phages to the amount of phages used for infection. One-step growth experiments were conducted based on modified methods described by Kitti et al. (2014). *A. baumannii* 15519 was infected by phage SH-Ab 15519 at an MOI of 0.005 and allowed to adsorb for 1 min at 37°C. Culture samples were taken every 2 min over a period of 26 min, and immediately assayed for plaque titer by the method described earlier. To determine the bacteriolytic activity of phage SH-Ab15519, *A. baumannii* 15519 was infected with phage SH-Ab 15519 at MOI of 0.01, 0.1, 1, or 10 at 37°C. Culture samples were collected at 30-min intervals for 5 h, and bacterial growth was measured based on OD_600_.

### Genomic Sequencing and Comparative Genomics Analysis

Genomic DNA of phage SH-Ab 15519 was sequenced using Illumina Hiseq 3000 (Illumina, San Diego, CA, United States). A total of 29,226,076 trimmed reads were obtained. The sequence assembly and optimization procedures were conducted by SOAPdenovo2 software (Luo et al., 2012). Possible open reading frames (ORFs) were predicted by GeneMark software (Besemer et al., 2001). Functional annotation of ORFs and homology assignments between genes from phage SH-Ab 15519 and other phages were performed by the BLASTp^1^. Alignment of functional proteins encoded by phages was conducted by Easyfig software (Sullivan et al., 2011). ORFs were searched by blast in virulence factor database (VFDB^2^) and antibiotic resistance gene database (ARDB^3^), to ensure its safety *in vivo*. The whole genome of phage SH-Ab15519 was deposited at GenBank under accession number KY082667.

### Effect of Phage Treatment against *A. baumannii* Infection in the Mouse Model

Seven-week-old BALB/c mice (female) were purchased from Shanghai Laboratory Animal Company (SLAC), China. Groups of 10 mice were rendered neutropenic via cyclophosphamide (Sigma, MO, United States) by intraperitoneal (i.p.) injection 4 days (200 mg/kg) and 1 day (150 mg/kg) before bacterial inoculation. The body weight and the neutrophil numbers were measured at designated time point before bacterial challenge to assess the neutropenic condition (Pantopoulou et al., 2007; Wang Y. et al., 2016). At day 0, the immunocompromised mice were anesthetized by i.p. injection with 2% pentobarbital (80 mg/kg), and then infected with *A. baumannii* strain 15519 at various concentrations by tracheal intubation. Survival curves of mice was monitored for 14 days. An inoculum of 10^8^ bacterial CFU was determined to be optimal and was used in the following experiments.

*Acinetobacter baumannii* strain 15519 was used for infection and phage SH-Ab15519 was applied as a therapeutic remedy. Groups of 10 mice were rendered neutropenic via cyclophosphamide injection as mentioned above, and infected with 10^8^ CFU of *A. baumannii* strain 15519 by tracheal intubation. Then a single dose of phage (30 μl, 10^7^, 10^8^, or 10^9^ PFU/mouse) or PBS was given intranasally at 1 hpi. To evaluate the time-dependent therapeutic efficacy, 10^9^ PFU phage or PBS was given intranasally at 1 or 2 hpi in groups of 12 mice, respectively. Survival curves were monitored for 14 days.

### Histopathology, Bacterial Burden, Phage Titer, and Cytokine Assay of Lung Tissue

For histological analysis, mice were rendered neutropenic via cyclophosphamide injection as mentioned above, and then divided into four groups: (i) control group, PBS given by tracheal intubation and PBS treated intranasally 1 hpi (*n* = 14); (ii) phage group, PBS given by tracheal intubation and phage (10^9^ PFU) treated intranasally 1 hpi (*n* = 14); (iii) Bacteria-infected group, *A. baumannii* strain 15519 (10^8^ CFU) infected by tracheal intubation and PBS treated intranasally 1 hpi (*n* = 14); (iv) phage-rescue group, *A. baumannii* strain 15519 (10^8^ CFU) infected by tracheal intubation and phage (10^9^ PFU) treated intranasally 1 hpi (*n* = 14). At 24 hpi, mice in each group were euthanized, and the lung tissues of 3 mice were removed and fixed in 4% paraformaldehyde, for embedment, slicing and hematoxylin and eosin (H&E) staining. The other six mice lung tissues were used to determine the phage titer through double-layer agar method and the bacterial burden using bacterial dilution-plate method. Bronchoalveolar lavage fluid (BALF) was collected from the other five mice, and TNF-α and IL-6 cytokines concentrations were detected by DuoSet enzyme-linked immunosorbent assay kits (R&D Systems).

### Statistical Analysis

Statistical calculations for mice survival rate were performed by Kaplan–Meier survival analysis with log-rank test, and the data of bacteria burden, phage count, and cytokine levels were analyzed statistically by Student’s *t*-test (GraphPad Prism 6.0 software). Results were considered statistically significant if *p* < 0.05.

### Ethics Statement

The conducts and procedures involved in the present work were approved by the Animal Ethics Committee of Shanghai Jiao Tong University School of Medicine.

## Results

### Antimicrobial Susceptibility Testing and Clonal Relationships of *A. baumannii* Strains

All 48 clinical isolates were resistant to ceftazidime, cefepime, piperacillin–tazobactam, and ciprofloxacin. The proportions of the isolates that were susceptible to tigecycline and colistin were 100.0 and 93.8%, respectively. The strains were resistant to most of other antimicrobial agents (**Table 1**). According to the MLST, 48 *A. baumannii* isolates were grouped into 18 distinct STs, including 10 existing STs, 2 unclassified STs, and 6 novel STs. ST1417 and ST1145 are the predominant STs, accounting for 29.2% (14/48) and 27.1% (13/48), respectively (Supplementary Table S1).

### Isolation and Morphology of *A. baumannii* Phages

**FIGURE 1:**
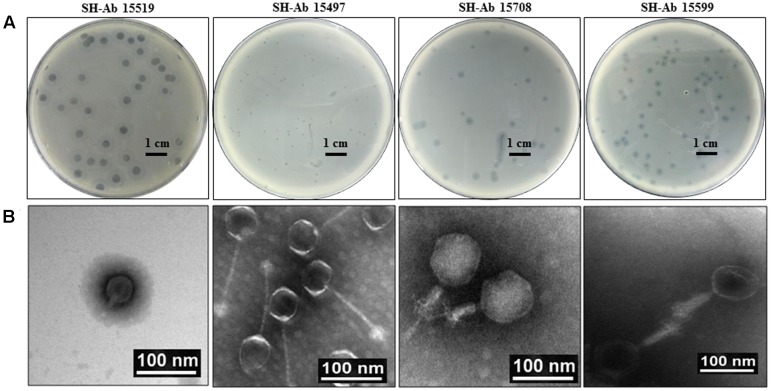
Plaque and TEM morphology of phages. **(A)** Plaque morphologies of phage SH-Ab 15519, SH-Ab 15497, SH-Ab 15708, and SH-Ab 15599. Scale bar, 1 cm. **(B)** TEM morphology of phage SH-Ab 15519, SH-Ab 15497, SH-Ab 15708, and SH-Ab 15599. Phages were negatively stained with potassium phosphotungstate. Scale bar, 100 nm.

About 30 different phages were isolated, and four phages with complementary host spectrums were chosen for further study. The four phages used in this study were designated as SH-Ab 15519, SH-Ab 15497, SH-Ab 15708, and SH-Ab 15599, respectively. Phage SH-Ab15519 formed clear, round plaques of 8–9 mm in diameter with significant haloes around (**Figure 1A**). Phage SH-Ab 15708 and SH-Ab 15599 formed clear plaques of 2-3 mm in diameter with haloes around while phage SH-Ab 15497 formed smaller (0.5 mm) plaques (**Figure 1A**). Transmission electron microscopy (TEM) revealed that phage SH-Ab15519 has a symmetrical polyhedral head and a short, straight, non-contractile tail (**Figure 1B**). The phage had a capsid of approximately 55 nm in diameter and a tail length of approximately 18 nm, thereby matching the typical morphological characteristics of *Podoviridae* family viruses (Ma et al., 2016). The following genomic analysis also supported this result. The tail of Phage SH-Ab 15497 was approximately 125 nm long by 4 nm wide and the head was about 55 nm in diameter. Phage SH-Ab 15708 showed a head of 88 nm long with a tail of 63 nm long while Phage SH-Ab 15599 showed a head of 88 nm long with a tail of 88 nm long (**Figure 1B**). Phage SH-Ab 15497 belongs to the family *Siphoviridae*. Phage SH-Ab 15708 and SH-Ab 15599 both belong to the family *Myoviridae* (**Figure 1B**).

### Lytic Spectrum of Single Phage and Phage Cocktail

Lytic spectrum was determined by spot test on 48 CRAB clinical isolates. The results showed that the lysis ranges of phage SH-Ab 15519, SH-Ab 15497, SH-Ab 15708, and SH-Ab 15599 were 16.6% (8/48), 29.2% (14/48), 29.2% (14/48), and 27.1% (13/48), respectively (Supplementary Table S1). The lysis range of phage cocktail was 87.5% (42/48), and no synergy was observed between phages in the cocktail (Supplementary Table S1). No correlation was detected between the ST type and host spectrum. Phage SH-Ab 15519 was chosen for further study because of its clear plaques and the size-enlarging haloes which suggested that this phage might produce depolymerase that degrades the exopolysaccharide of bacteria (Yan et al., 2014).

### Biological Characteristics and Lytic Effect of Phage SH-Ab 15519

**FIGURE 2:**
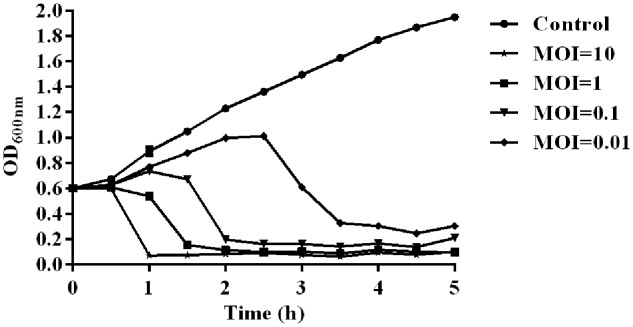
Lytic effect of phage SH-Ab15519 against *A. baumannii in vitro. A. baumannii* 15519 was infected by phage SH-Ab15519 at MOI of 0.01, 0.1, 1, or 10 and cultured for up to 5 h. *A. baumannii* 15519 cultured with the same volume of phage diluent was used as a control. This experiment was repeated three times, and the data were shown in the mean ± SEM.

The temperature and pH stability test revealed that phage SH-Ab 15519 is relatively stable at a temperature range from 4 to 50°C (Supplementary Figure S1A) and at pH between 5 and 12 (Supplementary Figure S1B). The absorption experiments illustrated that most phages (90%) were absorbed within 10 min (Supplementary Figure S1C). One-step growth experiments showed that the latent period of phage SH-Ab 15519 was 10 min and the burst size was 60 PFU per infected cell (Supplementary Figure S1D). The lytic activity of phage SH-Ab 15519 against *A. baumannii* 15519 was evaluated *in vitro*. The results showed that the growth of the *A. baumannii* was inhibited when co-cultured with phage in a concentration-dependent manner, with OD values declining more quickly at MOI 10 than at MOI 0.01, 0.1, or 1 (**Figure 2**).

### Genomic Analysis of Phage SH-Ab 15519

**FIGURE 3:**
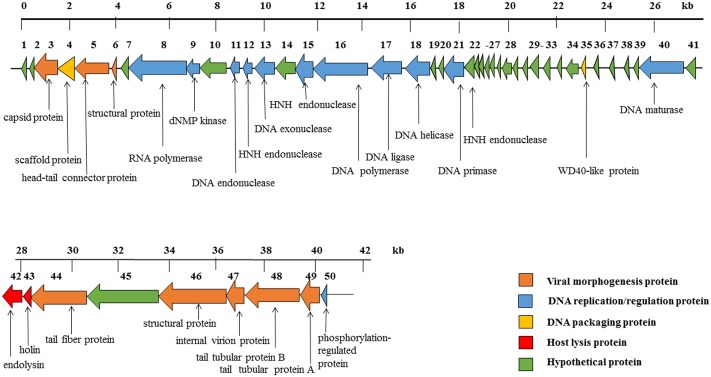
Diagram of phage SH-Ab15519 genome arrangement. Arrow indicates ORF, with different colors to illustrate groups of proteins with different functions.

The phage SH-Ab15519 genome is a linear, double-stranded DNA, with approximately 40,439 bp and a GC content of 39.5%. According to our TEM result, it belongs to the *Podoviridae* family, further confirmed by the absence of tape measure protein, which is responsible for tail assembly in *Myoviridae* and *Siphoviridae* families (Garcia-Heredia et al., 2013). The phage SH-Ab15519 genome encodes 50 predicted ORFs, with an average length of 745 bp. Proteins encoded by SH-Ab15519 could be classified into five groups: morphogenesis (eight proteins), DNA replication/regulation (12 proteins), DNA packaging (two proteins), host lysis (two proteins), hypothetical protein (26 proteins) (**Figure 3**). No virulence or antibiotic resistance genes were found in the genome when searched in the VFDB and ARDB database. In spite of similarities displayed by phage SH-Ab15519 with *Acinetobacter* phage vB_AbaP_PD-6a3 (GenBank accession number: KT388102.1; 93% coverage, 99% identity) and *Acinetobacter* phage phiAB6 (GenBank accession number: KT339321.1; 93% coverage, 96% identity) against NCBI database, SH-Ab15519 is a newly reported phage because it only shares limited completely identical proteins comparing with the other two phages, with the number of 22 and 1, respectively. And four specific proteins encoded by SH-Ab15519 (Supplementary Figure S2), such as, WD40-like protein facilitates protein–protein interactions and severs as scaffold to shape protein complexes (Gachomo et al., 2014), while calcium-binding tyrosine phosphorylation-regulated protein was once reported as fibrous sheath protein involved in capacitation (Naaby-Hansen et al., 2002). Apart from these specific proteins, there are quite a few proteins with several SNPs or indels among these phages showing marked differences between the three phages.

### Therapeutic Efficacy of Phage SH-Ab15519 against Mouse *A. baumannii* Lung Infection

The lung infection mouse model used in the present study was developed by intratracheal inoculation of *A. baumannii* in neutropenic mice. The body weight and neutrophil numbers dropped dramatically after two doses of cyclophosphamide infection (Jacobs et al., 2014; Takemura-Uchiyama et al., 2014; Supplementary Figure S3). The survival rates of mice with 10^9^, 10^8^, 10^7^, or 10^6^ CFU of *A. baumannii* 15519 inoculations were 18.18, 18.18, 36.36, and 90.91%, respectively (Supplementary Figure S4). Intratracheal application of 10^8^ CFU of *A. baumannii* was chosen to be the optimal dosage used in the following experiments.

**FIGURE 4:**
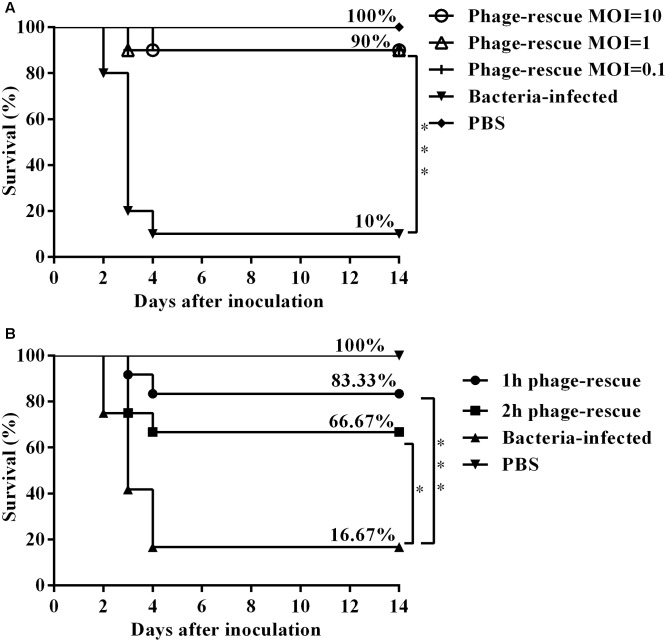
Therapeutic efficacy of phage SH-Ab15519 against mouse *A. baumannii* lung infections. Mice were rendered neutropenic via cyclophosphamide injection and then infected with 10^8^ CFU of *A. baumannii* strain 15519 by tracheal intubation. Survival rate was evaluated after intranasal administration of phage SH-Ab 15519 at different doses or different time post infection. **(A)** Dose-dependent analysis of therapeutic efficacy of phage SH-Ab 15519. Phages were intranasally inoculated with a dose of 10^7^ PFU (MOI = 0.1), 10^8^ PFU (MOI = 1), or 10^9^ PFU (MOI = 10) at 1 hpi (*n* = 10). **(B)** Time-dependent analysis of therapeutic efficacy of phage SH-Ab 15519. Dose of 10^9^ PFU (MOI = 10) Phages were intranasally administered at 1 or 2 hpi (*n* = 12). ^∗^*p* < 0.05; ^∗∗∗^*p* < 0.001.

To ascertain the effect of the phage SH-Ab15519 against bacterial infection, different doses were administered intranasally 1 hpi. Results showed that treatment with phage at an MOI of 0.1, 1, or 10 significantly increased the survival rate (90%) compared with the non-phage-treated control group (10%) (**Figure 4A**). Although different doses of bacteriophage treatment resulted in the same survival percentage, mice treated with bacteriophages in a MOI of 10 survived (one mouse died at 4 dpi) compared to the other phage treatment groups (one mouse died at 3 dpi) (**Figure 4A**). Mice in the group of MOI of 10 also had milder clinical signs (piloerection, lethargy, and obtuse movement) than the mice from other two phage treatment groups. So we chose a MOI of 10 as the standard dose in future experiments.

In time-dependent experiments, administering phage in MOI of 10 at 1 and 2 hpi resulted in survival rates of 83.33 and 66.67%, respectively (**Figure 4B**), which indicated that earlier administration of phage led to a better therapeutic efficacy.

### Histological Changes, Bacterial Burden, and Cytokine Analysis

**FIGURE 5:**
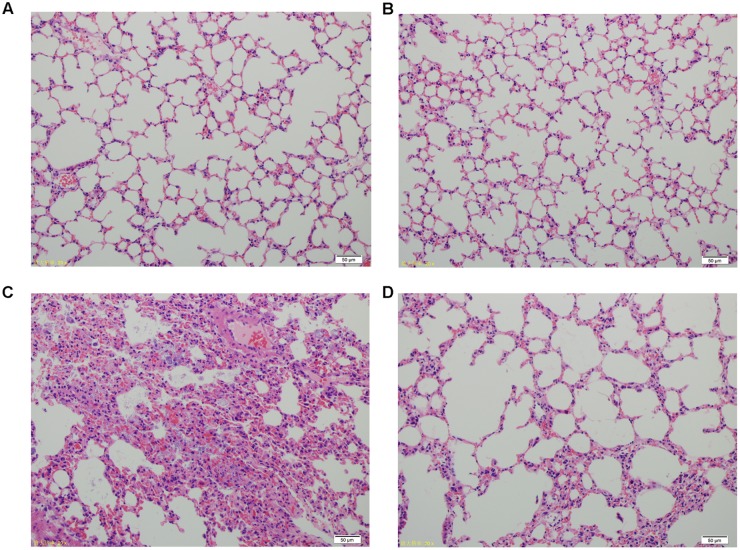
Histopathology of lung tissues from different groups of mice. **(A)** Control group. **(B)** Phage group, PBS given by tracheal intubation and phage (10^9^ PFU) treated intranasally 1 hpi. **(C)** Bacteria-infected group, *A. baumannii* strain 15519 (10^8^ CFU) infected by tracheal intubation and PBS treated intranasally 1 hpi. **(D)** Phage-rescue group, *A. baumannii* strain 15519 (10^8^ CFU) infected by tracheal intubation and phage (10^9^ PFU) treated intranasally 1 hpi. Lung tissues were removed at 24 hpi. H&E staining was used for histopathological examination. Magnification, ×200.

**FIGURE 6:**
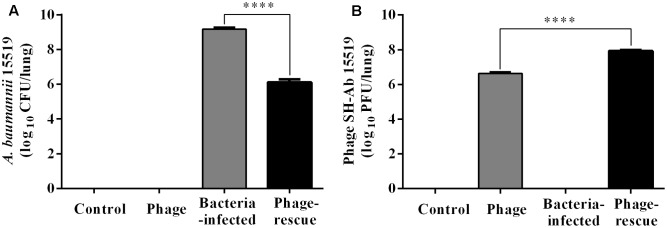
Bacterial burden and the phage titer in the lung at 24 hpi. **(A)** Mice in the bacteria-infected group and phage-rescue group were sacrificed at 24 hpi and the bacterial burden in lungs was measured by using bacterial dilution-plate method. **(B)** Mice in the phage-rescue group and phage group were sacrificed at 24 hpi and the phage titer in lungs was measured through double-layer agar method. The data were shown in the mean ± SEM (*n* = 6). ^∗∗∗∗^*p* < 0.0001.

**FIGURE 7:**
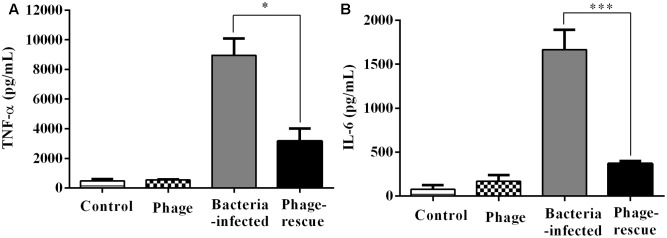
Levels of cytokines in the BALF of the different groups of mice. The BALF of the mice in the groups of control, phage, bacteria-infected, and phage-rescue were collected at 24 hpi. **(A)** TNF-α and **(B)** IL-6 concentrations of BALF were measured by ELISA. The data were shown in the mean ± SEM (*n* = 5). ^∗^*p* < 0.05; ^∗∗∗^*p* < 0.001.

Lung tissue from bacteria-infected group showed severe thickening and congestion of the alveolar walls and marked inflammatory cell infiltration in the perivascular and peribronchial areas at 24 hpi, while inflammation was greatly reduced in the phage-rescue group (**Figures 5C,D**). Moreover, the phage group showed no histological changes in the lung compared to the control group at 24 hpi (**Figures 5A,B**). The ability of phage SH-Ab 15519 to reduce bacterial burden in the lungs was also investigated (**Figure 6A**). Mice in the bacteria-infected group exhibited higher bacterial loads, reaching approximately 1.46 × 10^9^ CFU/lung, while the bacteria burden in phage-rescue group were decreased to 1.28 × 10^6^ CFU/lung at 24 hpi (**Figure 6A**). The phage titer in the lung tissues was also investigated. The results showed that 4.22 × 10^6^ PFU/lung phage titer was detected in phage group, and a higher phage titer was detected in phage-rescue group (8.30 × 10^7^ PFU/lung) (**Figure 6B**). Levels of the inflammatory cytokines, TNF-α and IL-6, in the BALF of the mice from different groups were measured at 24 hpi. The result showed that the levels of TNF-α and IL-6 were significantly elevated in the BALF of bacteria-infected mice, while phage treatment effectively reduced the excessive cytokines release caused by bacterial overgrowth (**Figures 7A,B**). The expression of cytokines remained at basal levels in the mice from both control and phage group (**Figures 7A,B**).

## Discussion

Recently, multidrug-resistant *A. baumannii* has emerged as one of the predominant organisms responsible for hospital-acquired pneumonia, wound infections and ventilator-associated pneumonia (Kollef et al., 2008; Atici et al., 2016). During an outbreak of CRAB in a hospital in Brazil (1999), 62.5% of the infected patients died and pneumonia was the leading cause of death (Dalla-Costa et al., 2003). The high mortality and morbidity of CRAB infections due to the limited option of antimicrobial agents necessitates the need for exploration of alternative therapeutic agents. Only recently, interest in phage therapeutics has been renewed and research in this area has been reinstated on a global scale. Bacteriophage treatments of animals, *ex vivo* human skin infections and surgical wound infections have been reported several times in many studies and these therapy outcomes are seen positively (Vieira et al., 2012; Regeimbal et al., 2016). In January 2017, Sarker and colleagues proved the safety and efficacy of oral application of *Escherichia coli* bacteriophage (Sarker et al., 2017). Furthermore in October 2017, Schooley and colleagues reported a case in which the personalized bacteriophage-based therapeutic cocktail was successfully applied intravenously to rescue a patient with severe disseminated resistant *A. baumannii* infection (Schooley et al., 2017). These clinical trials delineate the extent to which bacteriophage-based therapeutics could be used and provide a promising future for phage therapy. Mouse, mink, and primate lung infections models have been used for assessing the efficiencies of phage-based treatments but few of them is designed to rescue CRAB lung infections (Gu et al., 2016; Oduor et al., 2016). In the present study, we isolated a new bacteriophage, SH-Ab15519, which could effectively control lung infections that is caused by a clinical CRAB both *in vitro* and *in vivo*.

In this study, about 30 lytic phages against CRAB clinical isolates were isolated from hospital effluent, a good source of bacteriophages against pathogenic organisms. The host range of bacteriophage is usually narrow, which will impede their clinical usefulness. To tackle this problem, phage cocktails are used to broaden the *in vitro* lytic spectrum, and make it more effective than monophage therapy. Our study indicated that the combined infection spectrum of four phages was 87.5% (42 of 48) against CRAB clinical isolates, which represents a good coverage of CRAB strains. These phages are fabulous resources in the phage bank and are promising candidates for phage therapy in future investigations.

For the successful applications of phage as an alternative to traditional antibiotic therapy, novel phage discovery, complete genome analysis, and careful assessment of its therapeutic potential *in vivo* are essential before clinical application. Until now, 27 complete genomes of *A. baumannii* phages have been reported in the NCBI database^4^. However, only a few of these phages have been tested of their efficiency against CRAB infections *in vivo* (Jeon et al., 2016; Regeimbal et al., 2016). Based on the genome sequence, phage SH-Ab15519 is a novel phage that belongs to unclassified family *Podoviridae*. BLASTp analysis showed that the gene SH-Ab 15519_44 shares high similarity to the depolymerase of phage phiAB6 (Yan et al., 2014). Depolymerase is one kind of enzyme to degrade exopolysaccharide of bacteria, and has the potential to destroy bacterial biofilms and thus to render the bacterial more vulnerable to antibiotic drugs (Hatfull, 2008). The plaque morphology of phage SH-Ab15519 showed clear plaques with size-enlarging haloes, which is in accordance with the existence of depolymerase gene in phage SH-Ab15519. No virulence or antibiotic resistance genes were found in the genome of phage SH-Ab 15519 when searched in the VFDB and ARDB database, which implied the safety of phage *in vivo*. Biological characterization experiments showed that phage SH-Ab15519 exhibit significantly high stability at a broad pH range (4–12) and temperature range (4–50°C), which further supported its feasibility in storage and usage during phage therapy.

*Acinetobacter baumannii* mainly affects elderly patients with compromised immune systems and always leads to negative outcomes (Gaynes et al., 2005). The CRAB strain 15519 used in this study was isolated from a sputum sample of an 84-year-old male patient who died from severe pneumonia at Huashan Hospital in 2015. We used the well-characterized neutropenic murine pulmonary infection model to examine the therapeutic efficacy of phage SH-Ab15519 against CRAB. According to the study of Chiang et al. (2009), the interstitial inflammation in pulmonary parenchyma, regarded as early stage of bacterial pneumonia could be observed at 2 h after intratracheal inoculation of *A. baumannii*. In our study, phage SH-Ab15519 was applied at 1 or 2 h post-bacterial challenge to assess the *in vivo* phage therapeutic efficacy. The animal protection studies showed that phage administered intranasally can effectively rescue the mice from lethal *A. baumannii* lung infections, and significantly reduce bacterial burden and proinflammatory cytokines level in lung tissues. Meanwhile, histopathological examination also showed amelioration in bacterial lung infections with reduction in congestion and inflammatory cells infiltration after phage therapy. Our *in vivo* study demonstrated prominent efficiency of phage SH-Ab15519 treating CRAB strain medicated lung infections. The result of time-dependent experiments indicated that phage applied immediately after infection provide better protection in mice than delayed treatment, which is consistent with the previous study of Wang Y. et al. (2016). In their study, the intranasal phage therapy was applied to rescue mice from *A. baumannii*-mediated pneumonia and they found that the group in which phage was administered 1 h post-bacterial challenge was marked by a higher survival rate than the groups in which phage was applied 4 and 24 h post-bacterial challenge. These evidences imply that timely treatment is a critical factor determining phage therapeutic efficacy. However, similar high survival rates were defined among different phage MOI (0.1, 1, or 10) treatments (**Figure 4A**). In previous studies, the common amount of phage applied against bacterial lung infections was MOI 10, and lower MOI of 0.1 always resulted in dramatically lower survival rate (Cao et al., 2015; Jeon et al., 2016). The fact that phage SH-Ab15519 can effectively rescue the mice with low MOI (0.01) indicated its high efficiency to lyse the bacteria *in vivo*, which may be due to its short replication cycle (18 min) and the polysaccharide depolymerase it produced. More interestingly, in the phage group (phage applied without *A. baumannii* infection), a high phage titer (4.22 × 10^6^ PFU/lung) was still present in lung tissues 24 h after delivery. This was unexpected, because many studies reported that phages are rapidly eliminated from the body (Skurnik and Strauch, 2006). Previous studies showed that phages delivered by i.p. route or intravenous route were eliminated rapidly in the blood and organs of mice, leading to poor effect on infection prevention (Lu and Koeris, 2011). Our results indicated that phage therapy through intranasal routes is an appropriate way to treating bacterial lung infections which can provide high phage titer with long half-life in the target organ. Considering the property of high efficiency in clearing *A. baumannii* at low MOI, phage SH-Ab15519 may also have the potential of being used as prophylaxis to nosocomial CRAB pneumonia in ICUs (Chanishvili, 2016).

An earlier clinical trial showed that no adverse events were observed in phage therapy of pulmonary infections by nebulization (Kvachadze et al., 2011). Our study also implied the safety of phage SH-Ab15519 applied intranasally in mice. No obvious alterations were observed in lung tissue histology and proinflammatory cytokine levels in lung tissues of the phage group compared to the PBS group, which provided a further safety evidence for the phage therapy by intranasal route and supported the feasibility of phage therapy in respiratory infection.

In this study, while the *in vivo* phage efficacy was promising, it was an experimental “proof of *in vivo* bactericidal effect” that did not closely resemble clinical situation (such short an interval time is unlikely in clinical practice). Thus the ultimate applicability of phage SH-Ab15519 against *A. baumannii* infection still needs to be supported by clinical trials.

## Conclusion

Phage SH-Ab15519 is a novel *Acinetobacter* phage, which is considered safe for application in phage therapy based on complete genome analysis. Besides, our *in vivo* study demonstrated that phage SH-Ab15519 administered intranasally can effectively rescue the mice from lethal *A. baumannii* lung infections without deleterious side effects. Our work supported the potential use of lytic phages as an alternative therapeutic agent against infections caused by CRAB strains in the future.

## Author Contributions

FH and PH designed and supervised the study. YH, TL, and YY performed the experiments, analyzed the data, and drafted the manuscript. DD and RW did the experiments and analyzed the data. YW and MX performed the animal experiment. XG provided advice and suggestions.

## Conflict of Interest Statement

The authors declare that the research was conducted in the absence of any commercial or financial relationships that could be construed as a potential conflict of interest.

## Footnotes

1 http://blast.ncbi.nlm.nih.gov/Blast.cgi

2 http://www.mgc.ac.cn/VFs/main.htm

3 http://ardb.cbcb.umd.edu/

4 http://www.ncbi.nlm.nih.gov/pubmed, accessed March 15, 2017
