# Supplementary material for: Phage Therapy as a Promising New Treatment for Lung Infection Caused by Carbapenem-Resistant Acinetobacter baumannii in Mice
Source: Front Microbiol. 2018 Jan 9;8:2659. doi: 10.3389/fmicb.2017.02659 (PMC5767256; doi:10.3389/fmicb.2017.02659)
Supplement: Supplementary file 1 [file Presentation_1.PDF]

SUPPLEMENTAL MATERIAL

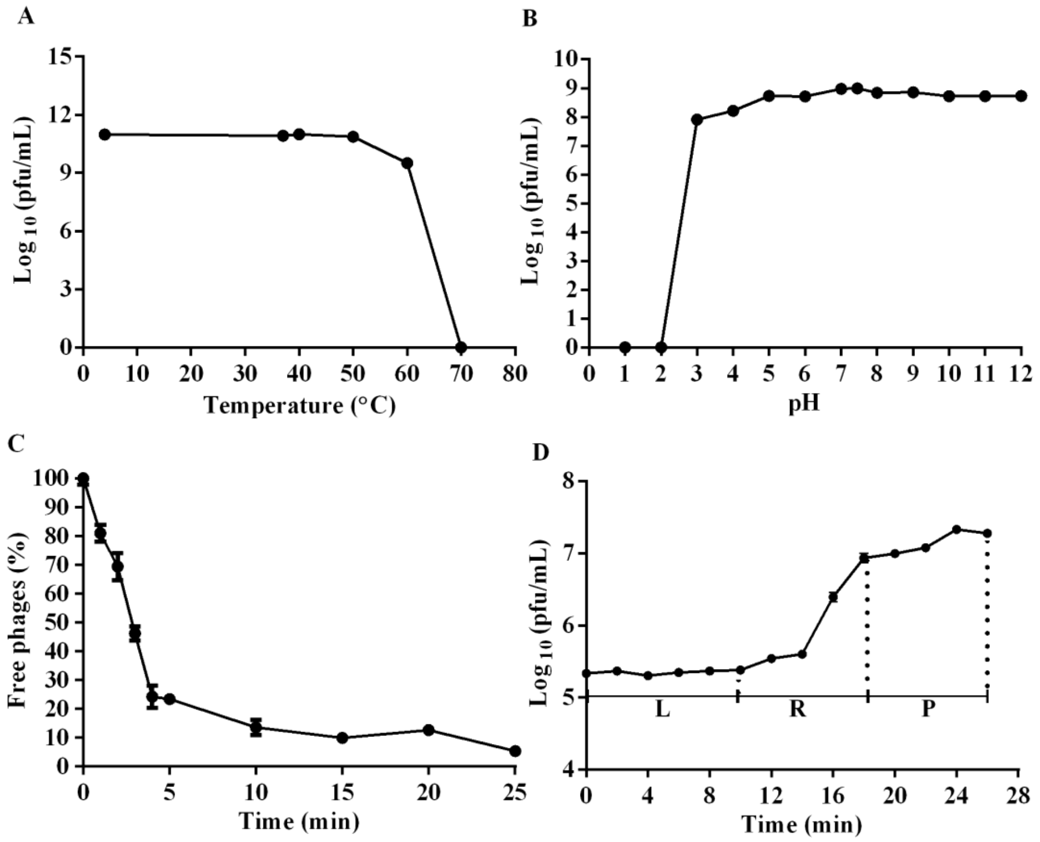

Figure S1 | Biological Characterization of phage SH-Ab15519.

(A) Stability of phage SH-Ab 15519 under different temperatures. **(B)** Stability of phage SH-Ab 15519 under different pH. **(C)** Adsorption of phage SH-Ab 15519 to *A. baumannii* 15519. The free phages proportion was the amount of non-adsorbed phages to the amount of phages used for infection. **(D)** One-step growth curve of phage SH-Ab 15519 on *A. baumannii* 15519. L: latent period; R: rise phase; P: platform phase. These experiments were repeated 3 times, and the data were shown in the mean ± SEM.

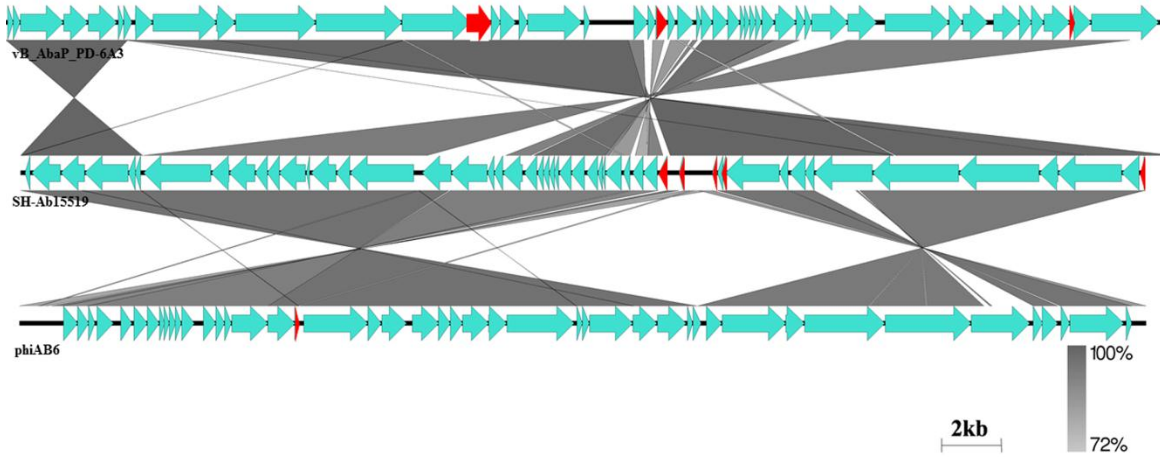

Figure S2 | The genome comparison of *A. baumannii* phage SH-Ab15519, *A. baumannii* phage vB_AbaP_PD-6a3 and *A. baumannii* phage phiAB6. Alignment of functional proteins encoded by phage SH-Ab15519 and *A. baumannii* phage vB_AbaP_PD-6A3 and *A. baumannii* phage phiAB6 was carried out using Easyfig software, with the direction of transcription illustrated by arrows. Proteins in red are encoded specifically by the phage. In left-to right reading, proteins specifically encoded by *A. baumannii* phage vB_AbaP_PD-6a3 (in superior line) all are hypothetical proteins; proteins specifically encoded by phage SH-Ab15519 (in middle line) are WD40-like protein, 3 hypothetical proteins and phosphorylation-regulated protein; protein specifically encoded by *A. baumannii* phage phiAB6 (in inferior line) is hypothetical protein.

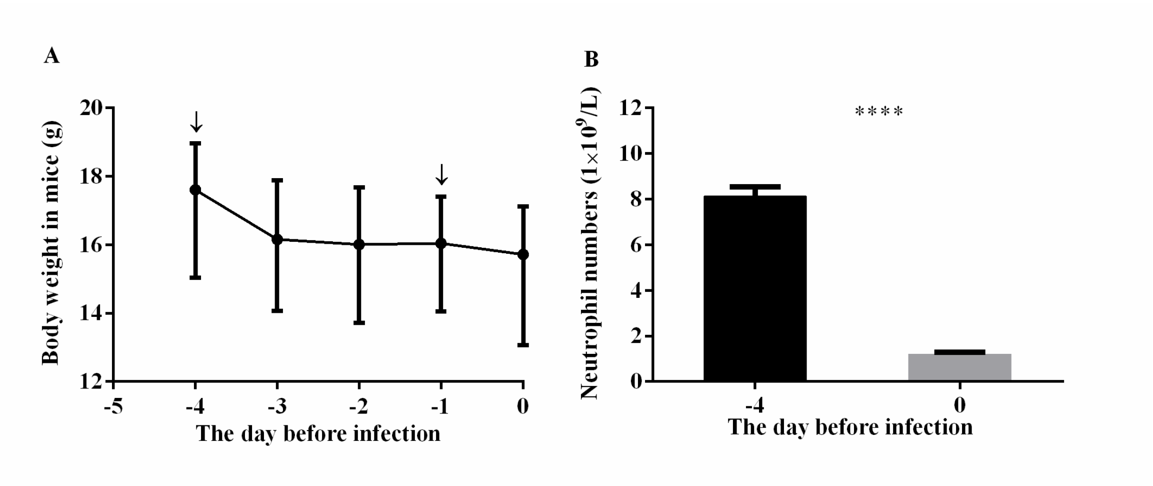

Figure S3 | Neutropenic mouse model. A mice model of *A. baumannii* AB15519 pneumonia was performed as previously described (Eveillard et al., 2010). Female BALB/c mice (7-week-old) were injected with cyclophosphamide at 4 days (200mg/kg) and 1 day (150mg/kg) before infection to construct neutropenia-mice model and mimic the clinical condition. The body weight and the neutrophil numbers were measured at designated time point. **(A)** Body weight curve. Mice were injected with cyclophosphamide at 4 days and 1 day (black arrows) before *A. baumannii* infection. Mice body weight was measured on 0, 1, 2, 3 and 4 days before infection. **(B)** Neutrophil change of cyclophosphamide pre-treated mice. On 0 and 4 days before infection, blood was collected and the WBC numbers were measured by Sysmex pocH-100iV Diff automatic animal blood analyzer (Sysmex, Hyogo, Japan). The data were shown in the mean ± SEM (n=30). ****, $p < 0.0001$.

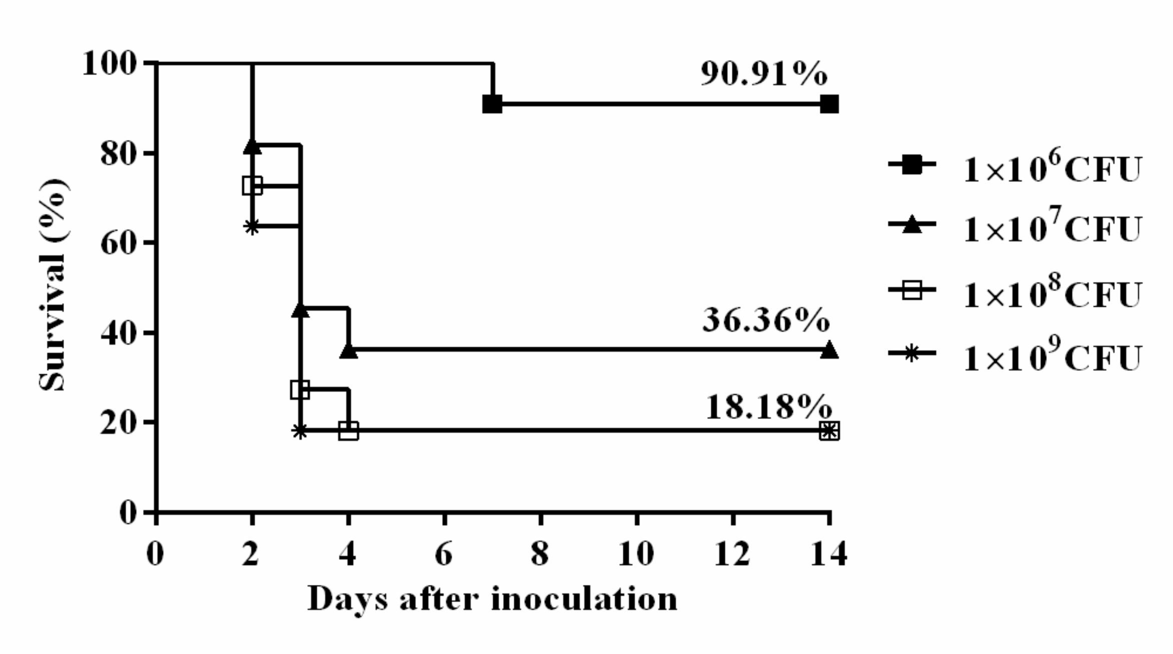

Figure S4 | Survival kinetics of mice infected with *A. baumannii* at various concentrations. The immunocompromised mice were anesthetized by an intraperitoneal injection with 2% pentobarbital (80 mg/kg), and then infected with 10^9, 10^8, 10^7 or 10^6 CFU of *A. baumannii* strain 15519 by tracheal intubation at day 0 as shown in Supplementary Figure S2A. Survival rate were monitored for 14 days (n=11).

Table S1 | The lysis activity of four phages and their cocktail against 48 *A. baumannii* clinical isolates

A. baumannii	**MLST**	**SH-Ab 15519 phage**	**SH-Ab 15497 phage**	**SH-Ab 15708 phage**	**SH-Ab 15599 phage**	**Phage cocktail**
979	1417	-	-	+	-	+
971	1145	+	-	-	+	+
959	1145	-	-	+	-	+
956	1417	-	-	+	-	+
915	1417	+	-	-	+	+
901	1145	-	-	+	-	+
894	new	-	-	+	-	+
889	368	-	-	+	-	+
884	208	-	-	-	-	-
878	381	-	+	-	-	+
855	new	-	-	+	-	+
839	1417	-	-	+	-	+
837	1144	-	+	-	-	+
818	1144	-	-	+	-	+
809	new	-	-	-	-	-
807	1417	-	-	-	-	-
752	new	-	-	+	-	+
737	new	-	+	-	-	+
732	1417	-	-	+	-	+
726	1145	-	+	-	-	+
725	1145	+	-	-	+	+
723	1417	-	+	-	-	+
716	/[a]	+	-	-	+	+
712	784	-	+	-	-	+
709	1417	-	-	+	-	+
708	784	-	-	+	-	+
705	1145	-	-	-	+	+
694	1417	-	+	-	-	+
683	1145	-	-	-	-	-
674	1145	-	+	-	-	+
654	451	-	-	-	+	+
653	1417	-	-	-	-	-
651	1417	-	+	-	-	+
605	556	-	-	-	-	-
599	/[a]	-	-	-	+	+
553	1144	-	-	+	-	+
551	1417	-	+	-	-	+
530	1417	+	-	-	+	+
520	1145	+	-	-	+	+
519	1145	+	-	-	+	+
507	1145	-	+	-	-	+
497	1145	-	+	-	-	+
496	540	-	+	-	-	+
494	1417	-	+	-	-	+
480	new	+	-	-	-	+
464	1145	-	-	-	+	+
458	540	-	-	-	+	+
456	368	-	-	-	+	+

[a] Unclassified sequence types. + :Susceptible - :Resistant
